# Efficacy of once-nightly sodium oxybate (FT218) in narcolepsy type 1 and type 2: post hoc analysis from the Phase 3 REST-ON Trial

**DOI:** 10.1093/sleep/zsad152

**Published:** 2023-05-29

**Authors:** Yves Dauvilliers, Thomas Roth, Richard Bogan, Michael J Thorpy, Anne Marie Morse, Asim Roy, Jordan Dubow, Jennifer Gudeman

**Affiliations:** Department of Neurology, Sleep-Wake Disorders Center, Gui-de-Chauliac Hospital, Institute for Neurosciences of Montpellier INM, INSERM, University of Montpellier, Montpellier, France; Sleep Disorders and Research Center, Henry Ford Health System, Detroit, MI, USA; University of South Carolina School of Medicine, Columbia, SC, USA; Medical University of SC, Charleston, SC, USA; Montefiore Medical Center, New York, NY, USA; Geisinger Commonwealth School of Medicine, Geisinger Medical Center, Janet Weis Children’s Hospital, Danville, PA, USA; Ohio Sleep Medicine Institute, Dublin, OH, USA; Avadel Pharmaceuticals, Chesterfield, MO, USA; Avadel Pharmaceuticals, Chesterfield, MO, USA

**Keywords:** extended release, narcolepsy, narcolepsy type 1, narcolepsy type 2, once-nightly dosing; sodium oxybate; randomized controlled trial

## Abstract

**Study Objectives:**

Post hoc analyses from the phase 3 REST-ON trial evaluated efficacy of extended-release once-nightly sodium oxybate (ON-SXB; FT218) vs placebo for daytime sleepiness and disrupted nighttime sleep in narcolepsy type 1 (NT1) and 2 (NT2).

**Methods:**

Participants were stratified by narcolepsy type and randomized 1:1 to ON-SXB (4.5 g, week 1; 6 g, weeks 2–3; 7.5 g, weeks 4–8; and 9 g, weeks 9–13) or placebo. Assessments included mean sleep latency on Maintenance of Wakefulness Test (MWT) and Clinical Global Impression-Improvement (CGI-I) rating (coprimary endpoints) and sleep stage shifts, nocturnal arousals, and patient-reported sleep quality, refreshing nature of sleep, and Epworth Sleepiness Scale (ESS) score (secondary endpoints) separately in NT1 and NT2 subgroups.

**Results:**

The modified intent-to-treat population comprised 190 participants (NT1, *n* = 145; NT2, *n* = 45). Significant improvements were demonstrated with ON-SXB vs placebo in sleep latency for NT1 (all doses, *p* < .001) and NT2 (6 and 9 g, *p* < .05) subgroups. Greater proportions of participants in both subgroups had CGI-I ratings of much/very much improved with ON-SXB vs placebo. Sleep stage shifts and sleep quality significantly improved in both subgroups (all doses vs placebo, *p* < .001). Significant improvements with all ON-SXB doses vs placebo in refreshing nature of sleep (*p* < .001), nocturnal arousals (*p* < .05), and ESS scores (*p* ≤ .001) were reported for NT1 with directional improvements for NT2.

**Conclusions:**

Clinically meaningful improvements of a single ON-SXB bedtime dose were shown for daytime sleepiness and DNS in NT1 and NT2, with less power for the limited NT2 subgroup.

## Graphical Abstract

**Figure:**
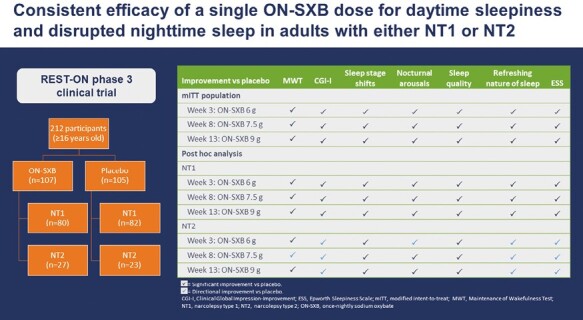

**Statement of Significance**

Narcolepsy is classified into two subtypes: narcolepsy type 1 (NT1), or narcolepsy with cataplexy, and narcolepsy type 2 (NT2), or narcolepsy without cataplexy. The etiology of NT2 is not well understood, and limited published data are available on the benefits of treatment specific to NT2, as trials have historically enrolled patients with narcolepsy with cataplexy or unspecified narcolepsy. A recently approved extended-release once-nightly sodium oxybate (ON-SXB) demonstrated effectiveness for treating excessive daytime sleepiness in adults with narcolepsy and cataplexy in patients with NT1. Results from post hoc analyses of the phase 3 REST-ON trial demonstrated consistent efficacy of ON-SXB in patients regardless of narcolepsy type. ON-SXB is the only once-at-bedtime oxybate treatment option for people with either narcolepsy type.

## Introduction

Narcolepsy is classified into two subtypes: narcolepsy type 1 (NT1), or narcolepsy with cataplexy, and narcolepsy type 2 (NT2), known as narcolepsy without cataplexy [1]. The distinction between narcolepsy with cataplexy and without cataplexy was first introduced in 2004 [2]. In 2014, the NT2 subtype was formally introduced into the *International Classification of Sleep Disorder* (3^rd^ Edition; ICSD-3) [3]. Patients with either NT1 or NT2 must have excessive daytime sleepiness (EDS), defined as “daily episodes of an irrepressible need to sleep or daytime lapses into sleep occurring for at least 3 months” in ICSD-3 [3, 4]. Cataplexy, or sudden muscle weakness in response to an emotion, is pathognomonic for a diagnosis of NT1 [3, 4]. However, cataplexy may be focal and mild or even atypical [5]; in some cases, patients may not recognize or relay this symptom, as they do not recognize it as related to narcolepsy [6].

The etiology of NT1 is recognized to be due to severe loss of hypothalamic hypocretin (orexin) neurons resulting in low or undetectable cerebrospinal fluid (CSF) orexin levels [5–7]. In contrast, the etiology of NT2 is not well understood; by definition, all patients diagnosed with NT2 have normal CSF orexin levels [5, 7–9]. However, 2 recent studies highlighted the existence of narcolepsy with intermediate CSF orexin levels, a very rare condition with a heterogeneous phenotype often including cataplexy [10, 11]. The additional symptoms that are part of the narcolepsy pentad—disrupted nighttime sleep (DNS), hypnagogic/hypnopompic hallucinations, and sleep paralysis—may be present in either narcolepsy type [4, 12]. Idiopathic hypersomnia (IH), which is also a central disorder of hypersomnolence, may differ from narcolepsy primarily due to the absence of 2 or more sudden onset REM periods (SOREMPs), as measured on the Multiple Sleep Latency Test (MSLT) [3]. IH may also be characterized by sleep inertia, long unrefreshing naps, and prolonged nighttime sleep [13]. Published literature has shown poor reproducibility of SOREMPs when repeated between participants diagnosed with NT2 and IH; a change in diagnosis upon repeat MSLT has been estimated to occur in 26% to >50% of patients initially diagnosed with NT2 [14, 15]. The proportion of patients with NT1/NT2/IH is difficult to accurately quantify from the literature; some publications identify NT1 as more prevalent, while others assert that NT2 is more prevalent [16], likely owing to the sometimes-subtle presentation of cataplexy and limited use of orexin measurements in clinical practice. The lack of reliability to differentiate NT2 and IH based upon MSLTs adds further complexity when estimating prevalence rates.

Guidelines in both the US and Europe recommend sodium oxybate (SXB, sodium salt of ɣ-hydroxybutyrate) for the treatment of adults with either NT1 or NT2 [17–19]. Two immediate-release oxybate formulations (SXB and calcium/magnesium/potassium/sodium [mixed-salt] oxybates) are currently approved in the United States for the treatment of cataplexy or EDS in narcolepsy and are dosed twice nightly [20, 21]; only immediate-release SXB is available in Europe [22]. The first dose of immediate-release oxybate is taken at bedtime, and the second dose is taken 2.5–4 h later [17, 23]. The short half-life (30–60 min) of ɣ-hydroxybutyrate [20] requires patients to awaken in the middle of the night for the second dose; as a result, sleep continuity can be disrupted to adhere to this dosing regimen [17, 23]. Patients with narcolepsy already experience fragmented sleep and poor sleep quality; thus, forced awakening for their second dose can further contribute to poor sleep quality [24–27].

The first once-at-bedtime oxybate (LUMRYZ, sodium oxybate for extended-release oral suspension [FT218; once-nightly sodium oxybate (ON-SXB)], Avadel Pharmaceuticals, Chesterfield, MO) was recently approved by the US Food & Drug Administration (FDA) for treatment of cataplexy or EDS in adults with narcolepsy [28]. In the pivotal phase 3 REST-ON trial, the efficacy and safety of ON-SXB were investigated in patients ≥16 years of age with NT1 or NT2. The 3 coprimary endpoints (mean sleep latency on the Maintenance of Wakefulness Test [MWT], much/very much improved rating on the Clinical Global Impression-Improvement [CGI-I], and the number of weekly cataplexy attacks) were significantly improved at weeks 3 (6 g), 8 (7.5 g), and 13 (9 g) (*p* < .001 vs placebo) [29]. Secondary endpoints were also statistically significant at all evaluated doses (*p* < .001 vs placebo), demonstrating decreases in the number of sleep stage shifts and nocturnal arousals and improvements in sleep quality, refreshing nature of sleep, and daytime sleepiness via the Epworth Sleepiness Scale (ESS) [29, 30]. Furthermore, ON-SXB was well tolerated among participants; common adverse drug reactions were those known to occur with SXB (nausea, vomiting, dizziness, enuresis, and headaches) and subsided over time [29].

There are limited published data on treatment efficacy in people with NT2 [5, 8, 9, 31]. In the 2021 American Academy of Sleep Medicine (AASM) Systematic Review, Meta-Analysis and GRADE publication [32], which accompanied the Clinical Practice Guidelines [19], the Task Force identified 6 randomized controlled trials (RCTs) of immediate-release SXB; 5 RCTs were limited to NT1, with 1 RCT in unspecified narcolepsy in which presence of cataplexy was not a requirement for enrollment [33]. Treatment with SXB has demonstrated improvement in EDS and cataplexy [32]. The objective of this post hoc analysis from the REST-ON trial was to evaluate the effect of ON-SXB on primary and secondary efficacy endpoint measures of EDS and DNS in separate subgroups of participants based on narcolepsy type.

## Methods

### Study Design

REST-ON was a multicenter phase 3, randomized, double-blind, placebo-controlled clinical trial (NCT02720744). The trial was designed to evaluate the efficacy and safety of ON-SXB for the treatment of narcolepsy. Full details on the trial design were previously published [29, 30]. Briefly, participants were randomized 1:1 to receive either ON-SXB or placebo; randomization was stratified by NT1 or NT2. After a 3-week screening period, there was a 13-week ON-SXB or placebo treatment period during which participants received 4.5 g for 1 week, 6 g during weeks 2 to 3, 7.5 g during weeks 4–8, and 9 g during weeks 9–13, dosed once nightly at bedtime. The trial ended with a 1-week follow-up period [29].

REST-ON was approved by institutional review boards at participating centers and conducted in compliance with the ethical principles of the Declaration of Helsinki, Good Clinical Practice guidelines, International Council for Harmonisation guidelines, and applicable national and local laws and regulatory requirements. Before participation in the study, participants provided written informed consent. For patients aged 16 or 17 years, consent was obtained from both the patient and their legal, authorized guardian [29].

### Participants

Full details on participants’ inclusion criteria were previously published [29, 30]. Eligible participants were ≥16 years of age with a diagnosis of NT1 or NT2 per ICSD-3 criteria, had continuing presence of EDS indicated by patient reports for the last 3 months, sleep latency <11 minutes on the MWT, and an ESS score >10. Participants diagnosed with NT1 must also have exhibited continuing cataplexy as indicated by an average of 8 attacks/week documented in the screening or baseline sleep and symptom diary [29]. Participants with prior SXB use were initially excluded from the study; a protocol amendment allowed for prior SXB use of ≤4.5 g per night for ≤2 weeks and ≥1 year before entering the study [30]. Participants diagnosed with sleep apnea or other sleep disorders known to cause EDS were excluded [30]. The use of concomitant stimulants was allowed during the study, provided participants were on a stable dose ≥3 weeks before the screening process and throughout the study.

### Assessment

This post hoc analysis evaluated primary and secondary endpoints from the REST-ON study in 2 separate subgroups: participants with NT1 and those with NT2. Coprimary efficacy endpoints were the change from baseline in MWT, CGI-I, and weekly cataplexy attacks (not applicable for this analysis). Secondary endpoints included change from baseline in the (1) number of sleep stage shifts, (2) number of nocturnal arousals, (3) patient-reported quality of sleep, (4) patient-reported refreshing nature of sleep, and (5) patient-reported ESS score. Both primary and secondary efficacy assessments were recorded at baseline and weeks 3 (6 g), 8 (7.5 g), and 13 (9 g).

On the test day, mean sleep latency on the MWT was averaged over five 30-minute trials. Each trial was terminated immediately after sleep onset or after 30 minutes if no sleep onset occurred [29]. CGI-I was the investigator-assessed rating of improvement in the overall condition compared to baseline (1, “very much improved,” to 7, “very much worse”). The number of sleep stage shifts was evaluated overnight using polysomnography (PSG). The number of shifts from stages N1, N2, N3, and rapid eye movement (REM) sleep to wake and from N2, N3, and REM sleep to N1 was recorded. Nocturnal arousals were assessed by the number of transient arousals on PSG as defined by the AASM scoring guidelines [34], as previously described [30]. Sleep quality and refreshing nature of sleep were documented daily on a 100-point visual analog scale (VAS). The VAS ranged from 1, representing “did not sleep”/“not refreshed,” to 100, representing “slept very well”/“refreshed.” VAS scores were collected using the daily sleep symptoms electronic diary and averaged over 14 days. Finally, the ESS [35] was used to evaluate subjective sleepiness in everyday situations. Participants rated their likelihood to fall asleep on a scale from 0 (never) to 3 (high) during 8 activities [29].

### Statistical Analyses

This post hoc analysis assessed the primary and secondary efficacy endpoints in the NT1 and NT2 cohorts separately and was conducted on the modified intent-to-treat population (mITT). The mITT population was composed of all participants with at least 1 efficacy measurement after receiving either 6 g of ON-SXB or placebo. Notably, the trial was not powered for subgroup analyses and enrollment of participants with NT2 was limited by the sample size of the NT1 population required for the cataplexy endpoint.

The change from baseline for mean sleep latency on the MWT with ON-SXB vs placebo at weeks 3 (6 g), 8 (7.5 g), and 13 (9 g) was analyzed using a mixed-effects model for repeated measures (MMRM). An MMRM model was also used to analyze changes from baseline with ON-SXB vs placebo at weeks 3 (6 g), 8 (7.5 g), and 13 (9 g) in (1) sleep stage shifts, (2) nocturnal arousals, (3) sleep quality, (4) refreshing nature of sleep, and (5) ESS score. In the model, treatment, visit, treatment by visit, time and treatment by time (ESS only), US or non-US study site, and baseline score were fixed effects; participants were random effects. The MMRM model also included covariate of baseline values and unstructured variance-covariance structure. GLIMMIX model for binomial data was used to analyze CGI-I categorized responses (ie, the proportion of participants who were very much or much improved), and the response variable was the observed values for each CGI-I categorized response [29]. The GLIMMIX model was composed of fixed effects, including treatment, visit, treatment by visit, US or non-US study site, and participants as random effects. Least-squares mean differences (LSMDs), odds ratios, 95% CIs, and *P* values were calculated. All statistical tests were performed using a two-sided α test with a 5% significance level.

## Results

### Participant Disposition and Demographics

As described previously, 222 participants were enrolled and randomly assigned to either ON-SXB or placebo (*n* = 111 each); 212 participants received ≥1 dose of the study drug [29]. Overall, 162 (76.4%) participants had NT1 (ON-SXB, 74.8%; placebo, 78.1%) and 50 (23.6%) participants had NT2 (ON-SXB, 25.2%; placebo, 21.9%) [30]. Baseline demographics, clinical characteristics, and measures of EDS and DNS were generally similar between treatment arms (Tables 1 and 2) [29]. The mean age of the population was 31.5 (range, 16–72) years [30]. Participants with NT1 were slightly older (mean age, 32.1 y) than those with NT2 (mean age, 28.3 y). There was a higher proportion of females in both the NT1 subgroup (72.8%) and the NT2 subgroup (52.0%), and the majority of the population was white (NT1, 76.5%; NT2, 72%). There were 190 participants included in the mITT population; 145 had NT1 (ON-SXB, *n* = 73; placebo, *n* = 72) and 45 had NT2 (ON-SXB, *n* = 24; placebo, *n* = 21).

**Table 1.** Baseline demographics and disease characteristics (safety population)

Characteristic	NT1		NT2	
	ON-SXB *n* = 80	Placebo *n* = 82	ON-SXB *n* = 27	Placebo *n* = 23
**Mean age (range), y**	32.1 (16–72)	32.2 (16–67)	27.4 (16–45)	29.3 (16–69)
**Median BMI (range), kg/m** ^**2**^	27.8 (17.7–71.9)	26.9 (18.1–46.5)	23.8 (16.9–37.0)	25.6 (18.8–43.2)
**Sex,** *n* **(%)**				
** Female**	55 (68.8)	63 (76.8)	14 (51.9)	12 (52.2)
** Male**	25 (31.3)	19 (23.2)	13 (48.1)	11 (47.8)
**Race,** *n* **(%)**				
** White**	62 (77.5)	62 (75.6)	18 (66.7)	18 (78.3)
** Black/African American**	15 (18.8)	14 (17.1)	6 (22.2)	1 (4.3)
** Asian**	1 (1.3)	5 (6.1)	2 (7.4)	3 (13.0)
** Other**	2 (2.5)	1 (1.2)	1 (3.7)	1 (4.3)
**Region,** *n* **(%)**				
** United States**	47 (58.8)	42 (51.2)	16 (59.3)	11 (47.8)
** Rest of world**	33 (41.3)	40 (48.8)	11 (40.7)	12 (52.2)

BMI, body mass index; NT1, narcolepsy type 1; NT2, narcolepsy type 2; ON-SXB, once-nightly sodium oxybate (FT218).

**Table 2.** Baseline measurements (mITT population)

	NT1		NT2	
Mean (SD)	ON-SXB *n* = 73	Placebo *n* = 72	ON-SXB *n* = 24	Placebo *n* = 21
**Primary endpoints**				
** MWT (min)**	5.0 (3.1)	4.7 (2.6)	5.0 (3.3)	4.9 (2.4)
** CGI-Severity**	5.3 (1.1)	5.2 (1.2)	4.8 (1.2)	4.6 (1.0)
**Secondary endpoints**				
** Number of sleep stage shifts**	62.1 (23.9)	60.9 (20.4)	53.9 (21.1)	58.0 (26.3)
** Number of nocturnal arousals**	82.8 (45.1)	80.3 (39.8)	79.0 (39.8)	66.5 (30.3)
** Sleep quality (VAS)**	52.3 (20.4)	56.8 (23.5)	58.4 (22.1)	52.8 (19.3)
** Refreshing nature of sleep (VAS)**	47.1 (20.7)	52.8 (24.2)	44.9 (25.3)	40.0 (16.6)
** ESS score**	17.3 (3.9)	17.9 (4.2)	14.5 (3.0)	16.4 (3.3)

ESS, Epworth Sleepiness Scale; mITT, modified intent-to-treat; NT1, narcolepsy type 1; NT2, narcolepsy type 2; ON-SXB, once-nightly sodium oxybate (FT218); VAS, visual analog scale.

### Efficacy

#### Coprimary Endpoints

##### MWT

**Figure 1:**
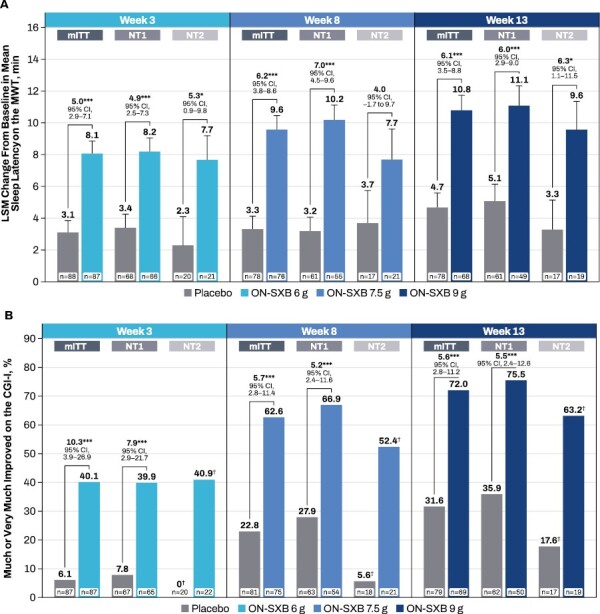
Improvement from baseline for the 2 coprimary endpoints by narcolepsy type (mITT population). (A) MWT (LSM change from baseline [MMRM analysis]) and (B) CGI-I (OR for “much” or “very much improved” [GLIMMIX model]). *p < .05, ***p < .001, ^†^Observed values reported. CGI-I, Clinical Global Impression-Improvement; LSM, least squares mean; mITT, modified intent-to-treat; MMRM, mixed model repeated measures; MWT, Maintenance of Wakefulness Test; NT1, narcolepsy type 1; NT2, narcolepsy type 2; ON-SXB, once-nightly sodium oxybate; OR, odds ratio.

At baseline, the mean (SD) sleep latency on the MWT was similar between the ON-SXB and placebo arms for both the NT1 and NT2 subgroups (Table 2). The least squares mean (LSM; [SE]) change from baseline in mean sleep latency at week 13 was 11.1 (1.1) min for ON-SXB 9 g vs 5.1 (1.1) min for placebo in participants with NTI (LSM difference vs placebo, *p* < .001 for) and was 9.6 (1.8) min vs 3.3 (1.9) min, respectively for NT2 (LSM difference vs placebo, *p* < .05; Figure 1A). In the NT1 subgroup, significant improvements were also observed at week 3 (LSM [SE] change from baseline in mean sleep latency: ON-SXB 6 g, 8.2 [0.9] min vs placebo, 3.4 [0.8] min; LSM difference vs placebo, *p* < .001) and week 8 (LSM [SE] change from baseline in mean sleep latency: ON-SXB 7.5 g, 10.2 [0.9] min vs placebo, 3.2 [0.9] min; LSM difference vs placebo, *p* < .001). In the NT2 subgroup, there was significant improvement with ON-SXB vs placebo at week 3 (6 g; LSM [SE] change from baseline in mean sleep latency: 7.7 [1.5] min vs 2.3 [1.6] min, respectively; LSM difference vs placebo, *p* < .05) and a numeric improvement that did not reach significance at week 8 (7.5 g; LSM [SE] change from baseline in mean sleep latency: 7.7 [1.9] min vs 3.7 [2.1] min, respectively).

##### CGI-I

The mean (SD) baseline CGI-Severity scores were similar between the ON-SXB and placebo arms for the NT1 and NT2 subgroups (Table 2). A significantly greater percentage of participants with NT1 receiving ON-SXB vs placebo were rated as “much” or “very much improved” on the CGI-I scale at week 3 (6 g; *p* < .001), week 8 (7.5 g; *p* < .001) and week 13 (9 g; *p* < .001; Figure 1B). Similarly, a greater percentage of participants with NT2 were “much” or “very much improved” on the CGI-I scale across all 3 ON-SXB doses vs placebo; however, the GLIMMIX model could not be calculated based on the small sample size.

#### Secondary Endpoints

##### Sleep Stage Shifts

**Figure 2.**
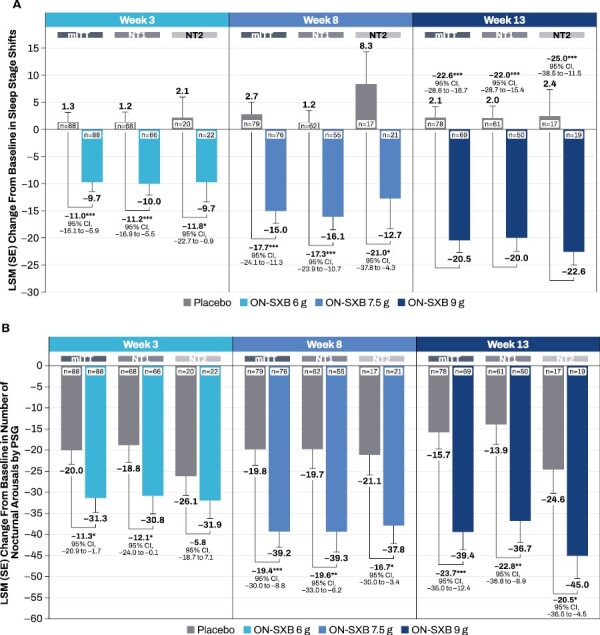

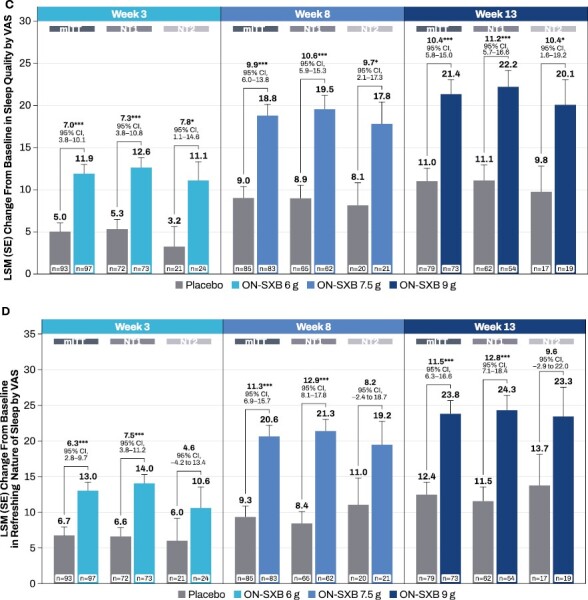

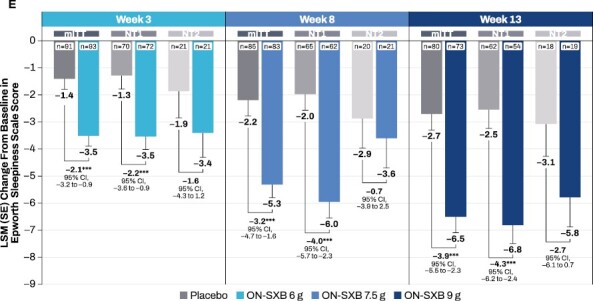
Change from baseline for the 5 secondary endpoints by narcolepsy type (mITT population [MMRM analysis]). (A) sleep stage shifts, (B) nocturnal arousals, (C) sleep quality, (D) refreshing nature of sleep, and (E) Epworth Sleepiness Scale. *p < .05, **p < .01, ***p ≤ .001. LSM, least squares mean; mITT, modified intent-to-treat; MMRM, mixed model repeated measures; NT1, narcolepsy type 1; NT2, narcolepsy type 2; ON-SXB, once-nightly sodium oxybate; PSG, polysomnography; VAS, visual analog scale.

At baseline, the total mean (SD) number of shifts from stages N1, N2, N3, and REM sleep to wake or from N2, N3, and REM sleep to N1 was similar between ON-SXB and placebo for the NT1 and NT2 subgroups (Table 2). The LSM (SE) change from baseline in the total number of sleep transitions for participants with NT1 was significantly reduced with ON-SXB vs placebo at week 3 (6 g; –10.0 [2.1] vs 1.2 [2.0]; LSM difference vs placebo, *p* < .001), 8 (7.5 g; –16.1 [2.4] vs 1.2 [2.3]; LSM difference vs placebo, *p* < .001) and week 13 (9 g; –20.0 [2.5] vs 2.0 [2.3]; LSM difference vs placebo, *p* < .001; Figure 2A). Similarly, participants with NT2 demonstrated significant reductions with ON-SXB vs placebo at week 3 (6 g; –9.7 [3.7] vs 2.1 [3.9]; LSM difference vs placebo, *p* < .05), week 8 (7.5 g; –12.7 [5.6] vs 8.3 [6.0]; LSM difference vs placebo, *p* < .05) and week 13 (9 g; –22.6 [4.6] vs 2.4 [4.9]; LSM difference vs placebo, *p* < .001).

##### Nocturnal Arousals

At baseline, the mean number (SD) of nocturnal arousals was similar between ON-SXB and placebo arm in participants with NT1 and NT2 (Table 2). The LSM (SE) change from baseline in the total number of nocturnal arousals was significantly reduced with ON-SXB vs placebo in the NT1 subgroup at week 3 (6 g; –30.8 [4.3] vs –18.8 [4.2]; LSM difference vs placebo, *p* < .05), week 8 (7.5 g; –39.3 [4.9] vs –19.7 [4.7]; LSM difference vs placebo, *P* < .01), and week 13 (9 g; –36.7 [5.2] vs –13.9 [4.8]; LSM difference vs placebo, *p* < .01; Figure 2B). Furthermore, statistically significant improvements in nocturnal arousals with ON-SXB vs placebo were observed in participants with NT2 at week 8 (7.5 g; –37.8 [4.4] vs –21.1 [4.8]; LSM difference vs placebo, *p* < .05) and week 13 (9 g; –45.0 [5.4] vs –24.6 [5.7]; LSM difference vs placebo, *p* < .05), but the reduction did not reach statistical significance at week 3 [6 g; –31.9 [4.3] vs –26.1 [4.6]).

##### Sleep Quality

At baseline, patient-reported mean (SD) sleep quality was similar between ON-SXB and placebo for both the NT1 and NT2 subgroups (Table 2). Participants with NT1 had improved LSM change from baseline (SE) in sleep quality with ON-SXB vs placebo at week 3 (6 g; 12.6 [1.2] vs 5.3 [1.2]; LSM difference vs placebo, *p* < .001), week 8 (7.5 g; 19.5 [1.7] vs 8.9 [1.6]; LSM difference vs placebo, *p* < .001), and week 13 (9 g; 22.2 [2.0] vs 11.1 [1.9]; LSM difference vs placebo, *p* < 0.001; Figure 2C). Similarly, improved sleep quality was observed with ON-SXB vs placebo in participants with NT2 at week 3 (6 g; LSM [SE] change from baseline: 11.1 [2.3] vs 3.2 [2.4]; LSM difference vs placebo, *p* < .05), week 8 (7.5 g; LSM [SE] change from baseline: 17.8 [2.6] vs 8.1 [2.7]; LSM difference vs placebo, *p* < .05), and week 13 (9 g; LSM [SE] change from baseline: 20.1 [3.0] vs 9.8 [3.1]; LSM difference vs placebo, *p* < .05).

##### Refreshing Nature of Sleep

Patient-reported mean (SD) rating on refreshing nature of sleep was similar at baseline between ON-SXB vs placebo in the NT1 and NT2 subgroups (Table 2). The LSM [SE] change from baseline in the refreshing nature of sleep was significantly improved from baseline with ON-SXB vs placebo in the NT1 subgroup at week 3 (6 g; 14.0 [1.3] vs 6.6 [1.3]; LSM difference vs placebo, *p* < .001), week 8 (7.5 g; 21.3 [1.7] vs 8.4 [1.7]; LSM difference vs placebo, *p* < .001), and week 13 (9 g; 24.3 [2.1] vs 11.5 [2.0]; LSM difference vs placebo, *p* < .001; Figure 2D). Participants with NT2 showed improvement from baseline in the rating of refreshing nature of sleep with ON-SXB vs placebo, but the differences did not reach statistical significance at any dose/time point (LSM [SE] change from baseline: 6 g, 10.6 [2.9] vs 6.0 [3.2]; 7.5 g, 19.2 [3.6] vs 11.0 [3.8]; 9 g, 23.3 [4.2] vs 13.7 [4.4]).

##### ESS

At baseline, mean (SD) ESS scores were similar between ON-SXB and placebo for participants with NT1 and NT2 (Table 2). The LSM [SE] change from baseline in ESS scores was significantly reduced with ON-SXB vs placebo in the NT1 subgroup at week 3 (6 g; –3.5 [0.5] vs –1.3 [0.5]; LSM difference vs placebo, *p* ≤ .001), week 8 (7.5 g; –6.0 [0.6] vs –2.0 [0.6]; LSM difference vs placebo, *p* < .001), and week 13 (9 g; –6.8 [0.7] vs –2.5 [0.7]; LSM difference vs placebo, *p* < .001; Figure 2E). Participants with NT2 had improved ESS scores from baseline with ON-SXB vs placebo at all 3 doses and time points, but the differences did not reach statistical significance (LSM [SE] change from baseline: 6 g, –3.4 [0.9] vs –1.9 [1.0]; 7.5 g, –3.6 [1.1] vs –2.9 [1.1]; 9 g, –5.8 [1.1] vs –3.1 [1.2]).

## Discussion

This post hoc analysis of data from REST-ON assessed efficacy endpoints in NT1 and NT2 subgroups separately and provides further insight into baseline measures of EDS and DNS in each narcolepsy type. Although patients with either NT1 or NT2 experience pathologic sleepiness [5], some clinicians believe that patients with NT2 may be less sleepy and some data show less sleep instability than those with NT1 [26]. However, baseline MWT and ESS values as well as factors associated with DNS (eg, sleep stage shifts, nocturnal arousals, sleep quality, and refreshing nature of sleep) were comparable between NT1 and NT2 subgroups in this analysis. These results also provide further support of the clinically significant improvement in objective and subjective symptoms of narcolepsy in adults with either NT1 or NT2 taking ON-SXB. Statistically significant improvement was demonstrated in MWT (coprimary endpoint) in patients treated with ON-SXB compared to placebo in the NT1 subgroup across all doses of ON-SXB, and significant improvement was demonstrated at 6-g and 9-g doses for the NT2 subgroup. For the CGI-I rating (coprimary endpoint), the NT1 subgroup had significantly more patients that were “much” or “very much” improved vs placebo at all time points. Owing to the small sample size in the NT2 subgroup, *p* values for the CGI-I endpoint could not be calculated; however, a numerically greater percentage of participants receiving ON-SXB vs placebo had a rating of “much” or “very much improved.” In addition, both subgroups had a significant change in the following secondary measures: (1) reduction in the number of transitions from sleep to wake and deeper stages of sleep to light sleep, (2) reduction in the number of nocturnal arousals, and (3) improvement in sleep quality. The patient-reported refreshing nature of sleep and ESS scores were statistically significantly improved with ON-SXB vs placebo in the NT1 subgroup. Improvements in these subjective secondary endpoints were numerically greater in the NT2 subgroup for ON-SXB vs placebo. Although not statistically different, likely owing to the limited NT2 sample, clinically significant improvement was achieved for both ESS and CGI-I (ie, improvement of 2 points on the ESS; 33% of patients with improvement on the CGI-I [pre/posttreatment difference]) [32]. This post hoc analysis in the NT1 and NT2 cohorts demonstrate clear alignment with the clinical efficacy observed from the primary REST-ON analysis for both primary and secondary endpoints. The sample size precludes the ability to make statistical comparisons between the groups; however, overall, these significant findings support the efficacy of ON-SXB in patients with NT1 or NT2 and importantly provides data specific to patients with NT2, which are currently limited in published literature. Moreover, these data underscore the importance of adequately treating NT2 symptomatology, particularly as the perception of less severe EDS and DNS is present in this subtype, which is contradicted by the baseline findings described in our study with strict inclusion criteria (eg, sleep latency <11 minutes on the MWT).

A diagnosis of NT1 can be made based on findings from CSF, however, lumbar puncture is rarely performed in the United States [36] and a worldwide survey found it was always done by only 1% of clinicians and rarely or never done by 76% [37]. NT2 is a heterogeneous condition that may be transient or stable with intermediate loss of orexin neurons and some overlap with NT1 and IH [6, 10, 11, 13]. More research is needed to understand the etiology of NT2 and the validity of the diagnosis over time as well as to further educate healthcare professionals and patients regarding the subtle manner in which cataplexy may present. Many experts recommend asking patients at routine visits if they are experiencing facial drooping, slurring of speech, or clumsiness, as these may be indicative of cataplexy [38]. Moreover, the sensitivity and specificity of the MSLT are less reliable for NT2 than for NT1, contributing to the challenges in diagnosing patients with NT2 [5, 6]. The limited reliability of the MSLT for diagnosing NT2 has been shown in multiple studies [14, 15, 39]. In one retrospective review, MSLT results were found to be reproducible for diagnosis of NT1 but not of NT2 [14]. Compared to controls, 26% of patients diagnosed with NT2 following MSLT had their diagnosis changed from narcolepsy to IH after the second MSLT [14]. In 2 other studies, a change in diagnosis over time occurred in over 50% of patients who were initially classified with NT2 after a second MSLT was conducted [15, 39]. As NT2 and IH may have similar symptomatology and are both central disorders of hypersomnolence, the poor reproducibility of the MSLT for NT2 makes differentiating NT2 from IH without long sleep time (LST) challenging [39].

When immediate-release SXB was initially approved by the FDA in 2002, the indication was limited to the treatment of cataplexy in narcolepsy. In 2005, the indication was expanded to include EDS in narcolepsy. In practice, some clinicians may reserve the use of oxybates for NT1, despite the approved indication [20, 21] and treatment guidelines [18, 19] recommending sodium oxybate for cataplexy or EDS. Additionally, no medication is FDA approved for the treatment of DNS in narcolepsy; however, ON-SXB has demonstrated improvement in both subjective and objective parameters of DNS [30], with this present analysis showing similar baseline values and clinical improvement in both NT1 and NT2 subgroups. Thus, although there has been a lack of studies examining the use of oxybates in NT2, and treatment efficacy data in people with NT2 in general, the current results add to the limited body of knowledge in people with this diagnosis and substantiate efficacy of ON-SXB for NT2 as well as NT1.

Overall, efficacy benefits with ON-SXB vs placebo were consistent between patients with NT1 and NT2. Although not all endpoints in the NT2 subgroup reached statistical significance, the magnitude of change was clinically significant. The lack of statistical significance in some of the secondary endpoints (ie, refreshing nature of sleep and ESS score) was likely a result of the small sample size of the NT2 subgroup and insufficient power; enrollment of participants with NT2 was limited by the oversampling of the NT1 group in REST-ON [29] to show the treatment efficacy on cataplexy. Given the variability of MSLTs in people with NT2, it is possible that repeat testing in participants with NT2 in REST-ON would result in some changes in diagnosis to IH without LST. Future research should further expand the knowledge base of treatment effects on NT2, including adequate power to evaluate endpoints specific to this subtype. Further studies should use the narcolepsy severity scale (NSS) to quantify the severity of symptoms such as disturbed nighttime sleep, sleep paralysis and hallucinations, and associated changes after taking medication [40].

ON-SXB was well tolerated in REST-ON. Adverse drug reactions during treatment with ON-SXB were consistent with the known safety profile of twice-nightly SXB and were generally mild or moderate [29]. The twice-nightly, immediate-release formulation of SXB has been FDA approved for twenty years [20]; once-at-bedtime ON-SXB may be a more suitable option for patients as less frequent dosing regimens are commonly associated with improved medication adherence [41–43]. This once-at-bedtime dosing covers a full night of sleep [44, 45], eliminating the need for patients to wake up for a second dose that could further contribute to DNS and poor sleep quality experience by people with narcolepsy [24, 25].

## Conclusions

The single bedtime dose of ON-SXB demonstrated clinically meaningful improvements in measures of EDS and DNS as well as overall condition compared to placebo in both the NT1 and NT2 subgroups across all doses evaluated (6, 7.5, and 9 g) in this post hoc analysis. In contrast to the twice-nightly oxybate formulations, ON-SXB does not disrupt or fragment sleep, and is less burdensome for patients as they can avoid the middle-of-the-night dose, which is particularly relevant given the potential chronic need for pharmacotherapy.

## Data Availability

The data underlying this article will be shared upon reasonable request to the corresponding author.

## Notes

**Clinical Trial Registration**: Once-Nightly Sodium Oxybate for Treatment of Excessive Daytime Sleepiness and Cataplexy in Narcolepsy; https://clinicaltrials.gov/ct2/show/NCT02720744; Registration ID: NCT02720744
